# Automated, flow-based chemiluminescence microarray immunoassay for the rapid multiplex detection of IgG antibodies to SARS-CoV-2 in human serum and plasma (CoVRapid CL-MIA)

**DOI:** 10.1007/s00216-021-03315-6

**Published:** 2021-05-13

**Authors:** Julia Klüpfel, Rosa Carolina Koros, Kerstin Dehne, Martin Ungerer, Silvia Würstle, Josef Mautner, Martin Feuerherd, Ulrike Protzer, Oliver Hayden, Martin Elsner, Michael Seidel

**Affiliations:** 1 Institute of Hydrochemistry, Chair of Analytical Chemistry and Water Chemistry, Technical University of Munich, Elisabeth-Winterhalter-Weg 6, 81377 Munich, Germany; 2 ISAR Bioscience GmbH, Semmelweisstr. 5, 82152 Planegg, Germany; 3 Helmholtz Zentrum München, German Research Center for Environmental Health, Haematologikum, Research Unit Gene Vectors and Technical University of Munich, Children’s Hospital, Marchioninistraße 25, 81377 Munich, Germany; 4 Institute of Virology, Technical University of Munich / Helmholtz Zentrum München, Trogerstr. 30, 81675 Munich, Germany; 5 German Center for Infection Research (DZIF), Munich partner site, 81675 Munich, Germany; 6 Heinz-Nixdorf-Chair for Biomedical Electronics, Technical University of Munich, TranslaTUM, Einsteinstr. 25, 81675 Munich, Germany

**Keywords:** SARS-CoV-2, COVID-19 serology, Flow-based chemiluminescence microarray immunoassay, Rapid multiplex antibody detection, Automated analysis platform

## Abstract

In the face of the COVID-19 pandemic, the need for rapid serological tests that allow multiplexing emerged, as antibody seropositivity can instruct about individual immunity after an infection with SARS-CoV-2 or after vaccination. As many commercial antibody tests are either time-consuming or tend to produce false negative or false positive results when only one antigen is considered, we developed an automated, flow-based chemiluminescence microarray immunoassay (CL-MIA) that allows for the detection of IgG antibodies to SARS-CoV-2 receptor-binding domain (RBD), spike protein (S1 fragment), and nucleocapsid protein (N) in human serum and plasma in less than 8 min. The CoVRapid CL-MIA was tested with a set of 65 SARS-CoV-2 serology positive or negative samples, resulting in 100% diagnostic specificity and 100% diagnostic sensitivity, thus even outcompeting commercial tests run on the same sample set. Additionally, the prospect of future quantitative assessments (i.e., quantifying the level of antibodies) was demonstrated. Due to the fully automated process, the test can easily be operated in hospitals, medical practices, or vaccination centers, offering a valuable tool for COVID-19 serosurveillance.

**Supplementary Information:**

The online version contains supplementary material available at 10.1007/s00216-021-03315-6.

**Figure:**
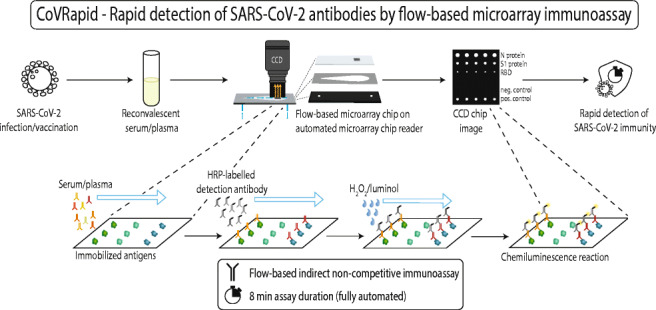
Graphical abstract

## Introduction

The global COVID-19 pandemic has kept the world in suspense for about a year now. The first cases of the novel coronavirus infection were reported in Wuhan, China, in December 2019 and assigned to the respective pathogen in January 2020 [1]. Since then, worldwide more than 90,000,000 people were infected with over 1,900,000 deaths resulting from COVID-19 (as of January 2021) [2].

The causative agent, SARS-CoV-2, is a betacoronavirus that is related to other zoonotic coronaviruses that circulate worldwide, causing common colds. SARS-CoV-2 has a large RNA genome, encoding for a number of structural proteins, namely the spike glycoprotein (S), the nucleocapsid protein (N), the membrane glycoprotein (M), and the envelope protein (E). Posttranslational modifications are essential for most of the proteins like the glycosylated membrane proteins and the phosphorylated N protein, which binds to viral genomic RNA [3]. In the course of a SARS-CoV-2 infection, the body reacts with the production of antibodies to a variety of these proteins, starting with IgM antibodies followed by the longer lasting IgG antibodies that can be found in the blood for several months after an infection. A very relevant factor in the body’s battle against the infection—and also for later immunity—is antibodies to the S protein, especially the receptor-binding domain (RBD) located in the S1 fragment. This domain binds to the human angiotensin-converting enzyme 2 (ACE2) and subsequently leads to the entry of the virus into the cell [4]. To assess whether an individual already underwent a SARS-CoV-2 infection and, hence, might be immune to reinfection due to protective antibodies, antibody tests are a helpful tool.

But these tests are not only relevant in context of previous infections. Since the emergence of SARS-CoV-2, a number of potential vaccine candidates have been developed [4] with the first ones already being applied [5]. Here, antibody tests might be helpful in testing the efficiency and the duration of immunity after vaccination. In addition, announcements have already been made (for example, by airlines) that access to certain locations and activities might be coupled to a proof of SARS-CoV-2 immunity. Here, rapid on-site antibody tests will be beneficial.

Therefore, we developed a chemiluminescence microarray immunoassay (CL-MIA) chip for the rapid, flow-based analysis of IgG antibodies to three different SARS-CoV-2 antigens—RBD, S1, and the N protein—in human serum and plasma in a fully automated analysis device, the Microarray Chip Reader 3. This device has previously been used for different tests, ranging from the quantification of bacteria by on-chip isothermal DNA amplification [6] over the detection of antibodies to viruses in pig blood [7] to the quantitative detection of antibiotic residues in milk [8] but here we present the first diagnostic application in human blood, the CoVRapid CL-MIA.

**Fig. 1:**
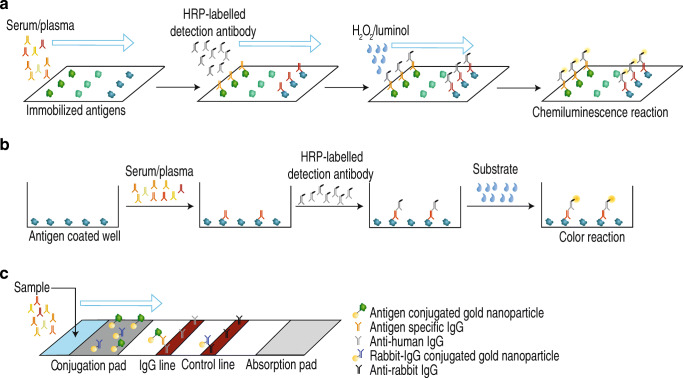
Overview over different antibody test principles. **a** Flow-based CL-MIA. **b** ELISA. **c** Lateral flow immunoassay

The test principle is an indirect non-competitive immunoassay that is carried out on microarray glass chips containing up to 100 covalently bound reagent spots per flow cell. The mode of operation is shown in Fig. 1 in comparison to other immunoassay techniques frequently applied for SARS-CoV-2 antibody detection. The flow-based principle of the CoVRapid test (Fig. 1a) allows for very short assay times below 10 min and is therefore even faster than many of the so-called rapid tests, which usually are lateral flow tests (Fig. 1c), and give qualitative results within 5 to 20 min [9]. These tests additionally have the disadvantage that they are sensitive to matrix effects resulting in relatively low sensitivity and the possibility of false positive results, which is undesired in the context of antibody testing [10]. Another relevant factor is the use of adsorbed, denatured antigens for most lateral flow assays that lack the three-dimensional structure that is relevant for the binding of neutralizing antibodies. In the CoVRapid CL-MIA, this crucial point can be accounted for, since the test uses native antigens from mammalian expression systems, containing all structural features and posttranslational modifications that are also present in antigens in infected human cells. A very specific kind of tests that is often used when quantitative high-throughput analysis is desired is ELISA tests (Fig. 1b). Here, antigen (often denatured) is adsorbed to wells and sample as well as labelled antibody and substrate are incubated within the wells. Therefore, many manual steps are necessary that might give rise to errors and prevent in-field applications, as extensive and expensive laboratory equipment is necessary. Additionally, since equilibrium conditions must be established, incubation times of usually several hours are needed prior to readout. Finally, only antibodies to one single antigen can be detected, meaning that false negative results would be obtained if patients formed antibodies to a different antigen that is not tested for [11]. This problem is overcome by microarrays that allow for multiplex analysis of various antigens, usually within hours [12]. Table 1 shows a comparative overview over different commercial SARS-CoV-2 antibody tests with respect to assay principle, assay time, tested antigens, and test performance. As is obvious from the information in Table 1, our CoVRapid test compares favorably and presents novelty in terms of work expenditure, multiplex capability, and assay time. Analysis is accomplished in a fully automated manner within 8 min giving information about antibodies to three different SARS-CoV-2 antigens simultaneously. The respective antigens are covalently immobilized in their native state using established coupling chemistry that can easily be applied to other proteins. This enables the expansion of the test to other antigens within a short development timeframe. The surface chemistry of the test, finally, also allows for negligible matrix influence, allowing even the analysis of hemolytic samples. With all these benefits and its high diagnostic sensitivity and specificity, the CoVRapid CL-MIA can be a valuable tool in COVID-19 serosurveillance.

**Table 1:** Overview over different assay principles and commercial SARS-CoV-2 antibody tests in comparison to CoVRapid CL-MIA (*n.s.*, not specified)

Assay principle	Test, *manufacturer*	Used antigen	Assay duration	Specificity in %	Sensitivity in %	Literature
Lateral flow assay	Panbio™ COVID-19 IgG/IgM Rapid Test Device, *Abbott Laboratories*	n.s.	10–20 min	99.4	93.0	[13]
	STANDARD™ Q COVID-19 IgM/IgG Duo Test, *SD Biosensor Inc*	N	15 min	100	64.9	[14]
Enzyme-linked immunosorbent assay (ELISA)	SARS-CoV-2 ELISA IgG, *EUROIMMUN AG*	S1	2 h	98	82.5	[14]
	EDI™ Novel Coronavirus COVID-19 IgG ELISA, *Epitope Diagnostics Inc*	N, S	< 2 h	98	85.6	[14]
Chemiluminescence immunoassay (CLIA)	MAGLUMI 2019-nCoV IgG, *Shenzhen New Industries Biomedical Engineering Co*	n.s.	n.s.	88.9–98	70.1–95.0	[14, 15]
	LIAISON® SARS-CoV-2 S1/S2 IgG, *DiaSorin S.p.A*	S1, S2	35 min	96.8–99	81.4–82.4	[14, 15]
	iFlash-SARS-CoV-2, *Shenzhen Yhlo Biotech Co. Ltd.*	N, S	> 12 min	92.9–100	76.9–93.0	[15, 16]
	SARS-CoV-2 IgG, *Abbott Laboratories*	N	29 min	99–100	64.5–92.6	[13, 14, 16]
	Elecsys® Anti-SARS-CoV-2, *Roche Diagnostics GmbH*	N	18 min	100	80.5–83.5	[14, 16]
Microarray immunoassay (MIA)	xMAP SARS-CoV-2 Multi-Antigen IgG Assay, *Luminex Corporation*	N, S1, RBD	2.5 h	99.3	96.3	[17, 18]
	CoVRapid CL-MIA, *Technical University of Munich*	N, S1, RBD	8 min	100	100	This work

## Experimental

### Chemicals, reagents and materials

All chemicals, unless stated otherwise, were purchased from Sigma-Aldrich, subsidiary of Merck (Darmstadt, Germany) and Carl Roth (Karlsruhe, Germany). Chemiluminescence reagents were used from the Elistar Supernova reagent kit from Cyanagen (Bologna, Italy). A peroxidase-labelled anti-human IgG antibody (Fc fragment) from goat was purchased from Sigma-Aldrich (A0170, 5.6 mg mL^−1^).

For the preparation of spotting, blocking, and running buffers, phosphate-buffered saline (PBS, 137 mM NaCl, 2.68 mM KCl, 8.09 mM Na_2_HPO_4_•2 H_2_O, and 1.47 mM KH_2_PO_4_, pH 7.2–7.4) was used. To obtain spotting buffer, 10% (w/v) trehalose dihydrate and 0.005% (w/v) Pluronic® F127 were added. For blocking buffer, 0.05% (*v*/*v*) Tween® 20 and 1% (w/v) bovine serum albumin were added to PBS. As running buffer, PBS with 0.1% (v/v) Tween® 20 was used.

### SARS-CoV-2 antigens

Recombinant SARS-CoV-2 spike S1 protein with mouse Fc-tag (expressed in HEK293 cells) was purchased from Biozol (Eching, Germany) and produced by Sino Biological (Beijing, China).

Recombinant SARS-CoV-2 spike RBD protein with His-tag and recombinant SARS-CoV-2 nucleocapsid protein with Strep-tag were produced by ISAR Bioscience (Planegg, Germany).

Spike protein RBD-His consists of the amino acids corresponding to the receptor-binding domain (RBD), which was derived from the S protein nucleotide sequence (positions 22517 to 23183, amino acid 319 to 541, RVQP….CVNF) of the SARS-CoV-2 Wuhan Hu-1 genome (GenBank accession number MN908947) followed by six histidines. Nucleocapsid protein N-strep consists of the amino acids corresponding to the N protein nucleotide sequence (positions 28290 to 29549) of the SARS-CoV-2 Wuhan Hu-1 genome (GenBank accession number MN908947) followed by a streptavidin tag (NP-Strep). The complementary DNA sequences adapted for hamster codon usage were produced synthetically by GeneArt (Life Technologies) by adding signal sequences METPAQLLFLLLLWLPDTTG before starting and cloned into the plasmid vector pcDNA5/FRT via BamHI and XhoI. The resulting vectors were called pcDNA5/CoV-RBD-His and pcDNA5/CoV-NP-Strep, respectively, and allow for expression and secretion of RBD-His or NP-Strep into the culture medium of mammalian cells under the control of the human cytomegalovirus (CMV) immediate-early enhancer/promoter and selection for stable clones with Hygromycin B after co-transfection with plasmid pOG44. The vectors were transfected by using Lipofectamine 2000 Reagent (Invitrogen, #11668-019) into Flip-InTM-Chinese hamster ovary (CHO) cells (Life Technologies), together with the plasmid pOG44, providing site-directed recombination. After selection of a stably expressing clone in Ham’s F12 supplemented with 10% fetal bovine serum and 600 μg ml^−1^ Hygromycin B, the clones were adapted to ProCHO5 medium (Lonza, #BE12-766Q) supplemented with 4 mM L-Glutamin (Biochrom, #K0283).

CHO-spike-RBD-His cells and CHO-spike-NP-Strep cells were grown in suspension in ProCHO5, 4 mM L-Glutamin and 600 μg ml^−1^ Hygromycin B in flasks to submaximal density at 37 °C and then centrifuged. The cells were continuously grown at 37 °C, with splitting every 3–4 days. The supernatants were cleared by centrifugation at 400*g* for 5 min and subsequent filtration with a 0.22-nm sterile filter (TPP, #99722). The resulting RBD-His or NP-Strep protein-containing medium was immediately frozen and stored at −20 °C until protein purification. Starting from a mix of cell clones, single clones are being selected and further propagated.

For protein purification, thawed CHO-RBD-His supernatants (0.5 L) were diluted 1:2 in 20 mM sodium phosphate, 0.3 M NaCl, pH 8.0, and loaded on an equilibrated 1-mL HisTrapTM excel column (GE Healthcare 17-3712-05). After washing the column with 20 mM sodium phosphate, 0.3 M NaCl, pH 8.0, RBD-His was eluted with 4 × 1 mL 20 mM sodium phosphate, 0.3 M NaCl, 0.25 M imidazole, pH 8.0. Protein content was determined by OD 280 measurement and the relevant fractions were dialysed (Slyde-A-Lyzer Dialysis Cassette, 10000 MWCO, Thermo Scientific # 66380) against phosphate-buffered saline (PBS from Roth: 137 mM NaCl, 2.7 mM KCl, 10 mM Na_2_HPO_4_, 2 mM KH_2_PO_4_, pH 7.4, 0.2 μm filtered and steam sterilized) at 4 °C for 16 h.

0.5 L CHO-NP-Strep supernatants were diluted 1:2 in 50 mM sodium phosphate, 0.3 M NaCl, pH 8.0, and loaded on an equilibrated 1-mL StrepTrapTM HP column (GE Healthcare 28-9075-46). After washing the column with 50 mM sodium phosphate, 0.3 M NaCl, pH 8.0, NP-Strep was eluted with 4 × 1 mL 20 mM sodium phosphate, 0.3 M NaCl, 2.5 mM desthiobiotin (Sigma, #D1411) pH 8.0. Protein content was determined by OD 280 measurement and the relevant fractions were dialysed (Slyde-A-Lyzer Dialysis Cassette, 10,000 MWCO, Thermo Scientific # 66380) against PBS at 4 °C for 16 h.

### Serum and plasma samples

Serum and plasma samples were either purchased from Sigma-Aldrich (Darmstadt, Germany) or obtained from Helmholtz Zentrum München, German Research Center for Environmental Health, Haematologikum (Munich, Germany) and the Institute of Virology, Technical University of Munich (Munich, Germany). All procedures were in accordance with the Helsinki Declaration of 1975, as revised in 2000.

All patient data were anonymized before obtainment of the samples. Patient samples were handled in laboratories approved for biosafety level 2.

### Chip surface chemistry

The immunoassay was performed on glass slides with surface modifications based on a procedure described elsewhere [19]. In short, microscopy glass slides were cleaned thoroughly and activated by acid treatment for subsequent silanization with (3-glycidyloxypropyl)trimethoxysilane. The silanized slides were then coated with Jeffamine® ED-2003. The prepared, polyether amine functionalized chips were stored under inert gas until protein immobilization was done.

### Microarray chip production

Depending on the immobilization protocol for antigen microarray preparation, the polyether amine functionalized glass slides were activated before spotting or used without activation. Activation was necessary for DSC and diepoxy PEG immobilization strategies, while for EDC/s-NHS immobilization, the functionalized glass slides could be used without further treatment.

For *N,N*′-disuccinimidyl carbonate (DSC) activation, a mixture of 16 mg *N,N*′-disuccinimidyl carbonate, 0.8 mg 4-(dimethylamino)pyridine, and 25 μL triethylamine in 320 μL dimethylformamide per chip was prepared. Subsequently, 600 μL of this mixture was pipetted onto the top side of a functionalized glass slide that was then covered with another slide (top side pointing downward). The chip sandwiches were incubated at RT and low humidity for 4 h, subsequently separated and sonicated in methanol for 15 min. After drying them in nitrogen stream, they were directly used for spotting.

For preparation of a reactive epoxy group on the chip surface, poly(ethylene glycol) diglycidyl ether (diepoxy PEG) activation was used. Therefore, 600 μL of diepoxy PEG was pipetted onto the top side of a functionalized glass slide that was then covered with another slide (top side pointing downward). The chip sandwiches were incubated at 100 °C overnight, subsequently separated and sonicated in methanol for 15 min. After drying them in nitrogen stream, they were directly used for spotting.

Alternatively, diepoxy PEG activation was done by pre-spotting the chips with a solution of diepoxy PEG in water (50% v/v) on the micro-contact spotter BioOdyssey Calligrapher® MiniArrayer from Bio-Rad (Hercules, USA) equipped with a solid pin SNS 9 from ArrayIT (Sunnyvale, USA). After pre-spotting and overnight incubation at 100 °C, the chips were also sonicated in methanol for 15 min and used for spotting after drying.

Spotting solutions were prepared by diluting the antigens and positive controls with spotting buffer for DSC and diepoxy PEG activated chips. For spotting without previous activation of the polyether diamine chip surface (EDC/s-NHS spotting), 2 mg/mL 1-ethyl-3-(3-dimethylaminopropyl)carbodiimide (EDC) and *N*-hydroxysulfosuccinimide (s-NHS) were added to spotting buffer. Antigen and positive control solutions of desired concentration (if necessary, previously diluted with spotting buffer) were then mixed with EDC/s-NHS solution (50% v/v). As positive control, anti-peroxidase and anti-human IgG antibodies were used, while as negative control spotting buffer was applied.

The spotting solutions were then pipetted into a 384-well plate (10–40 μL per solution depending on the number of spotted chips) and inserted into the micro-contact spotter together with the prepared glass chips. Spotting was done in five replicates for each spotting solution with a grid spacing of 900-μm distance between replicates and 1300-μm distance between the spotted rows. The spotting process was carried out at 20 °C and 55% humidity. After spotting, the chips were incubated at 20 °C and 55% humidity overnight.

For microarray chip assembly, the spotted chips were connected to a PMMA carrier containing in- and outlet holes using double-sided adhesive foil with cut-outs forming two flow channels. The assembled chips were then filled with blocking buffer and stored at 4 °C until measurement.

### Microarray measurements

Microarray measurements were carried out on the Microarray Chip Reader, 3rd generation (MCR 3), manufactured by GWK Präzisionstechnik GmbH (Munich).

Before the beginning of measurements on the microarray chip reader MCR 3, the system was flushed with running buffer and water using the respective flushing program. Subsequently, all necessary reagents (horseradish peroxidase (HRP)-labelled anti-human IgG diluted with running buffer to the desired concentration and chemiluminescence reagents luminol and hydrogen peroxide) were placed in the device. The tubes were loaded with the corresponding liquids using the load program. In the beginning of each measurement day, the blank program was executed to record the CCD camera background signal for an exposure time of 60 s. For measurements, a prepared microarray chip was inserted into the MCR 3 chip tray and the measurement program for the respective flow cell was carried out. Samples were prepared by diluting 100 μL of serum or plasma sample with running buffer to a final volume of 1 mL, out of which 900 μL was used for the measurement. The total assay process is summarized in Table 2. The sample was flown over the chip slowly, followed by the HRP-labelled detection antibody and the chemiluminescence reagents, which had been pre-mixed in 50 μL segments. The exposure time for the recording of the measurement image was 60 s, followed by thorough washing of the system, leading to a total measurement time of 7 min 45 s.

**Table 2:** Main assay steps on the MCR 3 with details to used volumes and flow rates; a video showing the measurement process is provided in the Supplementary Information

Step	Volume	Flow rate	
Sample injection	900 μL	10 μL s^−1^	
Flushing	1000 μL 2000 μL	10 μL s^−1^ 500 μL s^−1^	
Detection antibody injection	200 μL 800 μL	100 μL s^−1^ 10 μL s^−1^	
Flushing	1000 μL 2000 μL	10 μL s^−1^ 500 μL s^−1^	
CL reagents injection	400 μL	150 μL s^−1^	
Image acquisition	-	-	
Flushing of whole system	11 mL 8 mL	250 μL s^−1^ 500 μL s^−1^	(flushing of sample syringe) (flushing of tubes and chip)

### Data evaluation

The detected CL signals were corrected with the previously recorded blank image, stored as txt files and processed with the evaluation software MCR spot reader (Stefan Weißenberger, Munich, Germany). On the background-corrected CL images, a grid was set to define the position of the spots. For each spot, the mean value of the ten brightest pixels was calculated. Means and standard deviations were calculated for the five replicates per row and spots that deviated more than 10% from the mean were excluded (maximum two excluded spots per row).

The resulting mean values and standard deviations for all rows were used for further analysis and graphical evaluation using Python 3.

### Comparison measurements with commercial antibody tests

Comparison measurements with the commercial recomLine and recomWell tests from Mikrogen GmbH (Neuried, Germany) for the detection of SARS-CoV-2 specific IgG were conducted according to the manufacturer’s specifications.

## Results and discussion

### Optimization of immobilization strategy

**Fig. 2:**
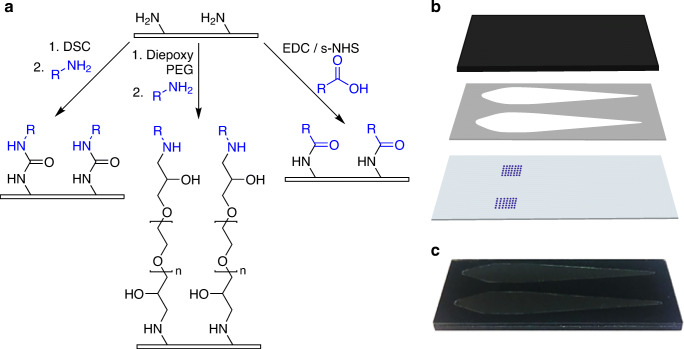
Microarray chip spotting and assembly. **a** Schematic overview of antigen immobilization strategies, with DSC and diepoxy PEG immobilization being two-step processes (chip surface activation followed by antigen immobilization) and EDC/s-NHS as one-step process (antigen activation in spotting solution), antigens shown in blue, immobilization is done via amino or carboxy groups of the amino acid side chains. **b** Chip assembly from carrier (top), adhesive foil with flow channels (middle), and glass microarray chip (bottom). **c** Photograph of an assembled chip

Four different methods of surface activation and antigen immobilization were tested (DSC, diepoxy PEG, diepoxy PEG pre-spotting, EDC/s-NHS). A schematic representation of the chemical background of each of these methods is presented in Fig. 2a) (for a more detailed view, Supplementary Information Fig. S1 shows the respective reaction schemes). The pre-functionalized microarray glass chips present PEG spacers with terminal amino groups on their surface. Antigens then can be immobilized in an undirected manner via either amino groups (e.g., from lysine) in DSC and diepoxy PEG immobilization or via carboxy groups (e.g., from glutamic acid) in EDC/s-NHS immobilization. For DSC, the full chip surface was activated, for diepoxy PEG activation of the full surface as well as only activation of the antigen spots by pre-spotting was tested. For EDC/s-NHS, the antigen carboxy groups are activated and spotted onto an amino functionalized chip without surface activation.

After spotting, the chips are assembled with a PMMA carrier and an adhesive foil containing two flow channels as shown in Fig. 2b), resulting in a microarray chip as in Fig. 2c) that can be inserted into the measurement device MCR 3.

**Fig. 3:**
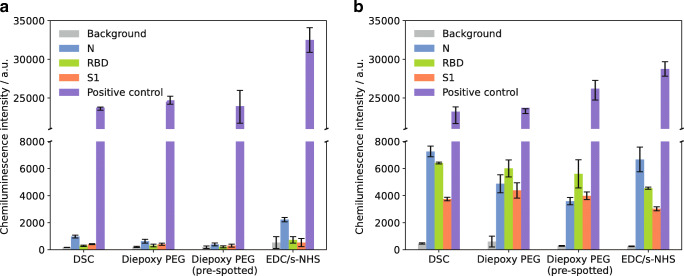
Measurement results for different immobilization methods. **a** SARS-CoV-2 serology negative sample. **b** SARS-CoV-2 serology positive sample; error bars represent replicate measurements on different chips, *n* = 3

Figure 3 shows the resulting chemiluminescence signals for measurements of a SARS-CoV-2 serology negative (a) and positive (b) sample for the SARS-CoV-2 antigens N, RBD, and S1, as well as the positive control (anti-human IgG) and the background signal (spotting buffer).

With all tested methods, the antigen CL signals were higher for the positive sample compared to the negative one, while for the background a very low signal and for the positive control a high signal were found, showing the general applicability of all methods. Pre-spotting of diepoxy PEG gave similar results as whole chip activation with diepoxy PEG with slightly lower signal for the positive sample. Therefore, the time-consuming pre-spotting process was considered unnecessary.

With the negative sample in Fig. 3a, only slight unspecific binding of antibodies to the antigens could be seen for DSC and diepoxy PEG, while for EDC/s-NHS especially for the N protein a relatively high signal was found (2231 a.u. compared to 630 a.u. for diepoxy PEG). This unspecific binding also diminishes the obtained positive/negative signal ratio, which is found as 3.0 (N), 6.3 (RBD), and 5.7 (S1) for EDC/s-NHS, while diepoxy PEG immobilization gave values of 7.7, 19.3, and 10.7 and DSC yielded 7.4, 21.9, and 9.1, respectively. Additionally, many of the EDC/s-NHS spots on the microarray chip were very variable, while the other immobilization methods gave uniform, round spots. As in EDC/s-NHS activation not the chip surface but carboxy groups of the proteins in solution are activated, cross-linking of the proteins might occur, leading to conformational changes and a change of activity over the course of the spotting process.

As the positive/negative signal ratios obtained with DSC and diepoxy PEG immobilization were comparable for all spotted rows, it was decided to use DSC immobilization for all further experiments due to the low time expenditure of 4 h for the surface activation before spotting compared to overnight activation with diepoxy PEG.

We therefore were able to develop different strategies for the covalent immobilization of proteins on glass microarray chips in their native conformation, benefitting from the expertise of our research group in the production of different kinds of microarrays. An important factor is the spotting buffer, containing trehalose and Pluronic® F127 [19]. Trehalose is also used in protein freeze-drying processes as a protective agent, mimicking the hydrogen bonds between polar functional groups of the protein and water [20], while pluronics are poloxamers that are widely applied in pharmaceutical industry and microfluidic technology as non-ionic surfactants to prevent protein aggregation and adsorption [21, 22]. The immobilization methods can also be easily applied to other native proteins, allowing for the rapid adaption and extension of the microarray.

### Optimization of antibody and antigen concentrations

**Fig. 4:**
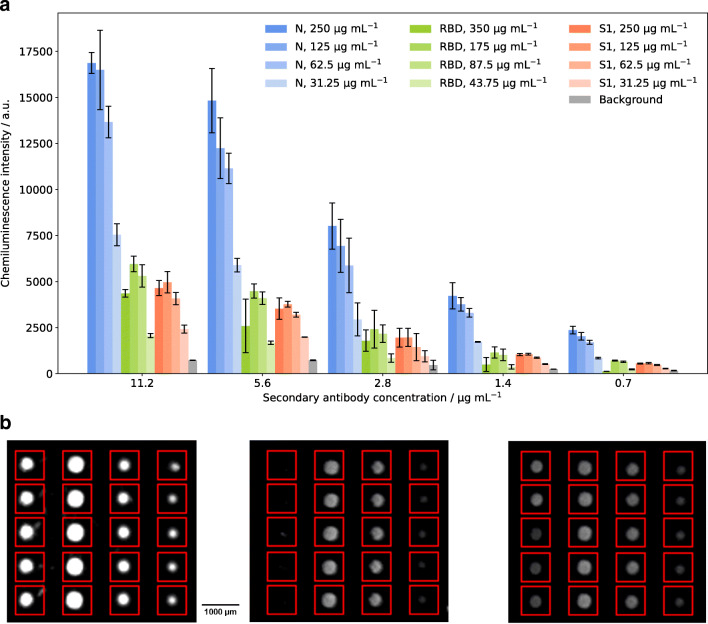
**a** Measurements of different immobilized antigen dilutions using different secondary antibody concentrations; error bars represent replicate measurements on different chips, *n* = 3. **b** Exemplary chip images (columns from left to right: N undiluted, N 1:2, N 1:4, N 1:8; RBD undiluted, RBD 1:2, RBD 1:4, RBD 1:8; S1 undiluted, S1 1:2, S1 1:4, S1 1:8, rows represent replicates of the same antigen dilution)

After determination of the optimal immobilization method, different immobilized antigen concentrations and secondary antibody concentrations were tested. For the antigen concentrations, undiluted antigen (250 μg mL^−1^ for N and S1 protein, 350 μg mL^−1^ for RBD) and subsequent twofold dilutions were tested until a dilution of 1:8. For the HRP-labelled secondary antibody, five different concentrations, namely 11.2 μg mL^−1^ (1:500 dilution of stock solution), 5.6 μg mL^−1^ (1:1000), 2.8 μg mL^−1^ (1:2000), 1.4 μg mL^−1^ (1:4000), and 0.7 μg mL^−1^ (1:8000), were used. The same SARS-CoV-2 serology positive sample was used for all measurements. The results are presented in Fig. 4a with one set of bars for each secondary antibody concentration and each bar representing a certain antigen concentration in the spotting solution as indicated. Figure 4b shows examples of images of a microarray chip with bright spots in rows representing spotted antigens.

It is clearly visible that the chemiluminescence signal increases with increasing secondary antibody concentration. In the same course, the background signal increases but to a lower extent compared to the specific antigen signals. For secondary antibody concentrations from 0.7 to 5.6 mg mL^−1^, a significant increase can be seen upon doubling of the concentration, while a further increase to 11.2 mg mL^−1^ only gives slightly higher signals for all antigens. Thus, as a compromise between high signal intensities and low expenditure of secondary antibody, a concentration of 5.6 mg mL^−1^ was used for all further measurements.

For the decision on the optimal spotted antigen concentration, not only the chemiluminescence intensities as displayed in Fig. 4a were taken into account but also the appearance of the spots on the microarray chip as shown in Fig. 4b. Here, three blocks of spots can be seen with the four columns within each block representing the different concentrations of antigens, decreasing from left to right. The three blocks correspond to the different antigens, starting with N protein on the left side, RBD in the middle and S1 protein on the right-hand side, as also shown in Fig. 4a. The flow direction of sample and reagents during the measurements is from lower towards higher concentrations of the antigens (right to left in Fig. 4b).

For the intensities, the same trends can be seen regardless which secondary antibody concentration was used. For the N protein, a signal increase is seen with increasing concentration of antigen on the chip. For S1 and RBD, this is true for all diluted samples (concentrations between 31.25 and 175 μg mL^−1^ as indicated above). For the spotting of undiluted antigen, lower intensities than for the 1:2 dilution are found, indicating either a lower immobilization efficiency with less immobilized antigen molecules on the surface or a lower activity of the protein. When looking at the antigen spots, an increase in diameter is seen up to the 1:2 dilution, while for the undiluted spotting row, notably smaller and less uniform spots are seen for all antigens, especially for the S1 protein, where the spots are barely visible. This can be attributed to the drying of the spots during the incubation time after spotting. In the undiluted antigen samples, no stabilizing agents were added, leading to rapid drying of the spots and activity loss of the protein. Especially for the S protein, it has already been shown that antibody recognition depends strongly on the used protein expression systems; therefore, also slight conformational changes upon drying of the spots might have an influence [3]. Additionally, it is possible that protein agglomerated or adsorbed to the wells of the microwell plate before spotting, reducing the concentration on each spot. For the diluted antigen samples, we aimed at reducing these effects by using the spotting buffer containing trehalose and Pluronic® F127. As these additives had a beneficial effect on signal intensity (for S1 and RBD) as well as on spot appearance (for all antigens) but still a high antigen concentration was desired, all further experiments were done using 1:2 dilutions of the antigens with spotting buffer, resulting in concentrations of 175 μg mL^−1^ (RBD) and 125 μg mL^−1^ (S1, N) in the spotting solution.

### Dilution measurements

To evaluate the correlation between antibody concentration and chemiluminescence signal that is needed for the development of prospective future quantitative tests, COVID-19 reconvalescent plasma was diluted with a negative control sample. Antibody measurements of samples with positive plasma ratios between 0 and 100% were performed for RBD and S1 protein as they are the most immunogenic antigens [3] and, therefore, most promising for a quantitative application. A determination of the SARS-CoV-2 N protein was not attempted, as its sequence was shown to be more conserved over different corona viruses [11] implying that cross reactivity and, hence, cross sensitivity to antibodies to endemic corona viruses might be possible [23].

**Fig. 5:**
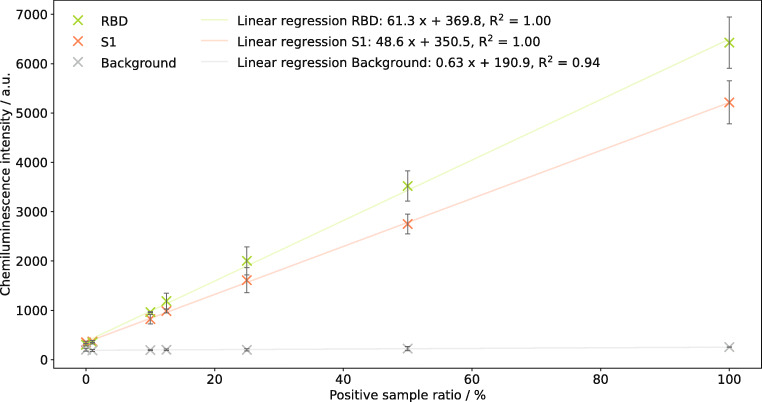
Linear regression (*m* = 7) for measurements of samples with different ratios of SARS-CoV-2 serology positive plasma for RBD and S1 protein; error bars represent replicate measurements on different chips, *n* = 3

Figure 5 shows the resulting chemiluminescence intensities for seven different mixture ratios of serology positive and negative samples. Besides the RBD and S1 protein, also the background signal is shown for comparison. A linear correlation between antibody concentration in the sample and chemiluminescence intensity can clearly be seen with linear regressions almost perfectly fitting the measured data (*R*^2^ = 1.00 for RBD and S1, *R*^2^ = 0.94 for the background signal). The slight slope for the background signal can be explained as different blood samples were used, naturally resulting in different background values. For prospective future applications, this matrix influence can easily be avoided by a background correction of the measurement data as was done in all following experiments.

While for the measurement point at 1% positive sample ratio only a slight difference to the pure negative sample can be seen, the following measurement point at 10% positive sample ratio already can be distinguished well from the negative sample. The greater slope for the RBD compared to the S1 protein can be assigned to the different spotted concentrations (175 μg mL^−1^ and 125 μg mL^−1^, respectively). From these results, we conclude that a future development towards a quantitative test is possible. If a standard sample with a known, high concentration of antibodies to the RBD and S1 protein is available, a calibration of the test can be done, allowing for a simple quantitative interpretation of measurements that might give a more detailed information about an individual’s SARS-CoV-2 serological status.

### Measurement and classification of patient samples and comparison with results of commercial antibody tests

To define cutoff values used for the assignment of positive and negative results, receiver operating characteristic (ROC) curves were used. They illustrate the trade-off between correctly identified positive samples and false positives in a diagnostic test, allowing for the selection of a suitable cutoff value for a given question [24]. In a ROC curve, the cutoff value is shifted over a range of values and sensitivity and specificity are calculated for each cutoff. Resulting pairs of sensitivity and 1 − specificity are plotted together with a diagonal line (*x* = *y*). A perfect test will result in a right triangle that intersects the point [0,1], representing 100% sensitivity and specificity. A calculation of the area under the curve (AUC) for a perfect test will give a value of 1.0, while the worst possible result (resembling a toss coin) is an AUC of 0.5, achieved by a ROC curve matching the diagonal. Depending on the diagnostic question of interest, a cutoff can be chosen with respect to highest possible sensitivity or specificity. In the context with SARS-CoV-2 antibody detection, high specificity is desirable, as a false positive result might mislead tested individuals to be less cautious as they presume to be immune to SARS-CoV-2 infection.

**Fig. 6:**
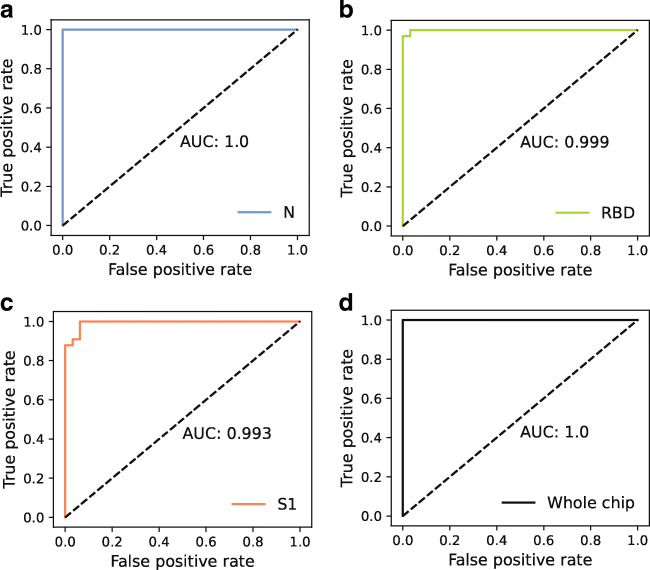
ROC curves and respective AUC values for different antigens, obtained from measurements of 65 patient samples; **a** N protein, **b** RBD, **c** S1 protein, **d** combination of all antigens on the chip

To determine ROC curves for the test presented herein, 65 serum and plasma samples (32 from individuals without previous SARS-CoV-2 infection, 33 from reconvalescent COVID-19 patients) were tested, the resulting chemiluminescence values were background-corrected and then used for the ROC determination. Results for the three tested antigens as well as for the combination of them on the chip are shown in Fig. 6. For the combined antigens, a sample was considered positive when it gave signal above cutoff for at least one antigen.

For all antigens, a high AUC above 0.99 was determined. Literature states that an AUC value above 0.9 represents good accuracy of a test [24, 25]. Especially for the N protein, an optimal ROC curve with an AUC of 1.0 was found.

Cutoff values were defined such that the highest possible specificities resulted for each antigen as especially in antibody testing, a false positive result is considered more harmful than a false negative one as it dissembles a non-existing immunity. The respective cutoff values, given in background-corrected chemiluminescence intensity, are 2860 for the N protein (100% sensitivity, 100% specificity), 800 for RBD (93.9% sensitivity, 100% specificity), and 1700 for the S1 protein (87.9% sensitivity, 100% specificity). Still, the given values for sensitivity must be considered with caution, as it cannot be guaranteed that all reconvalescent patients actually had formed antibodies to all antigens.

**Fig. 7:**
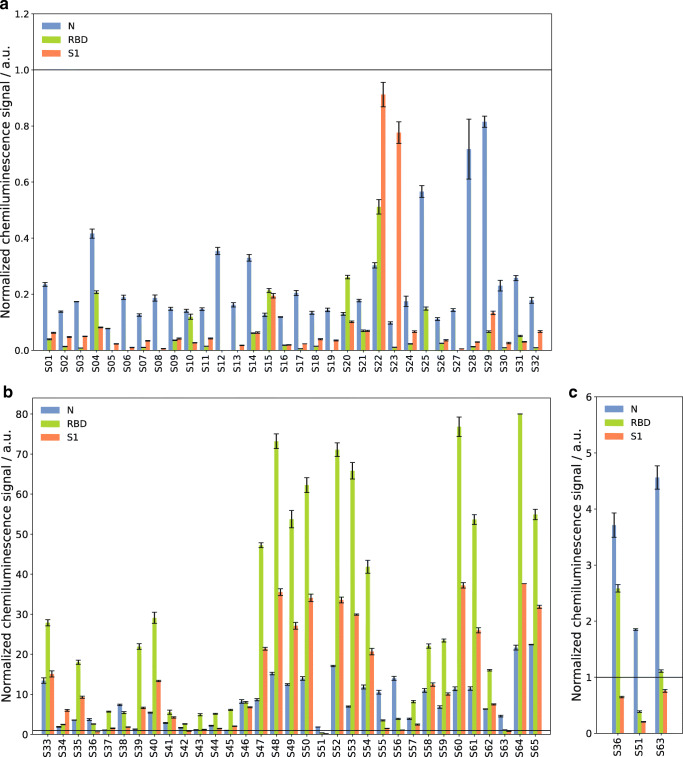
CoVRapid CL-MIA results for 65 patient samples. **a** Results for 32 SARS-CoV-2 serology negative samples. **b** Results for 33 SARS-CoV-2 serology positive samples. **c** Detailed representation of positive samples with measurement signal below the cutoff for at least one antigen; all values are normalized with respect to the cutoff values determined by ROC curve analysis; error bars represent standard deviation of replicate spots on one chip, *n* = 5

The determined cutoff values were then used to take a closer look at the measurement results for all patient samples. The background-corrected chemiluminescence values were normalized with respect to the cutoff values and the resulting values are shown in Fig. 7a for the negative samples and in Fig. 7b and c for the positive samples. For the patients without previous SARS-CoV-2 infection, most samples showed higher normalized intensities for the N protein than for RBD and S1. This was the expected result, as for the N protein a cross reactivity with endemic coronaviruses could not completely be excluded due to high sequence similarity. Comparison measurements with the recomLine test from Mikrogen showed that the vast majority of all patients had formed antibodies to the N protein of at least one of the endemic coronaviruses 229E, NL63, OC43, and HKU1. For the negative sample S22, which shows a lower intensity for N than for S1 and RBD, it is possible that a recent, undetected infection with SARS-CoV-2 was present and IgG antibodies had already started to form to a low extent or that the patient had overcome COVID-19 at a very early stage of the pandemic and the antibody amount in the blood had already declined below the detectable level. Confirmation would be possible by follow-up measurements of the patient or by consulting his case file, which both were not possible due to the sample obtainment strategy. Sample S23 shows a low intensity for both N protein and RBD, while a relatively high signal for S1 is detected. As the RBD is contained within the S1 protein and tended to give higher signals compared to the S1 in our test, this indicates that no specific antibodies to SARS-CoV-2 S1 have been formed. Instead, as the used S1 protein carried a mouse Fc fragment, it might be possible that the patient had formed human anti-mouse antibodies (HAMA) that have been shown to interfere with immunoassay measurements [26]. Still, with the defined cutoff values, all negative samples were correctly classified as negative for all three antigens, resembling a specificity of 100% for the CoVRapid CL-MIA.

For the SARS-CoV-2 serology positive samples in Fig. 7b the trend already seen in the dilution measurements again is visible, as for most patients a higher intensity is found for RBD compared to S1 due to the higher immobilized concentration. Few samples show a different behaviour with comparable intensities for S1 and RBD or even higher signal for S1, indicating that antibodies to other S1 regions than the RBD might have been formed. In comparison to the signals for the N protein, most samples show higher intensities for S1 and RBD, which is expected as the spike protein is considered more immunogenic than the nucleocapsid [3].

The measured intensities spread over a broad range from slightly above 1 (cutoff) to over 70. As no information on the clinical course of the patients was available, it can only be suspected that higher intensities may be related to either more recent or more severe disease. Still, all knowingly positive samples were found positive for at least one of the tested antigens, resembling 100% sensitivity.

For a total of three of the positive samples only for one or two of the antigens, a signal above the cutoff was determined. As in Fig. 7b no clear interpretation of samples with low signal is possible, these samples are shown in more detail in Fig. 7c. The reason for this outcome might be that the patients still were in an early stage of infection where few antibodies had been formed yet, or that the antibody amount in the blood was already declining due to a prolonged time since infection. This emphasizes that a quantitative test will be helpful in the future. When comparing the results obtained with the commercial multiplex test recomLine from Mikrogen, for S51 and S63, only a positive result for the N protein could be found, while S1 and RBD were negative, confirming the CoVRapid result. This is also in accordance with literature findings showing that antibodies to different proteins form independently which possibly leads to significantly different reactions to different antigens at certain points of time after symptom onset [27, 28].

Comparison tests were done not only with the recomLine test (N, RBD, and S1 protein) but also with the N specific recomWell ELISA from Mikrogen. The principal antibody test used for the sample classification that Fig. 7 refers to was the iFlash test from YHLO. Here, samples are classified with regard to antibodies for either the N or S1 protein.

Overall, a good performance of all tests was found as depicted in Table 3. While our CoVRapid test classified all samples correctly (with respect to iFlash classification), with the recomLine test, one false positive sample was found (positive for S1, negative for RBD and N). With the recomWell test, two samples gave results in the borderline area and were therefore excluded. Additionally, this test gave one false negative and one false positive result. This gives the CoVRapid test the highest sensitivity and specificity (100% each) while recomLine obtained values of 100% and 96.8%, respectively, and recomWell showed the highest deviations with 96.7% each.

**Table 3:**
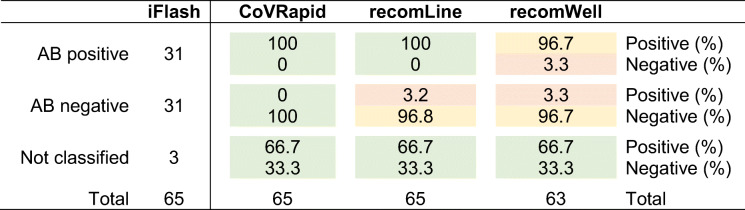
Classification of patient samples by different anti-SARS-CoV-2 IgG tests. The iFlash-SARS-CoV-2 IgG was used as reference classification for 62 samples, while three samples were only tested with the alternative tests CoVRapid CL-MIA, recomLine SARS-CoV-2 IgG, and recomWell SARS-CoV-2 IgG (one of the samples specified as “Not classified” was ordered from a commercial supplier as negative control, two were obtained from reconvalescent COVID-19 patients). For the recomWell test, two samples gave values in the borderline area of the test and were therefore excluded

## Conclusion

We developed a rapid, flow-based CL-MIA that allows for the fully automated detection of IgG antibodies to three different SARS-CoV-2 antigens, namely N, S1, and RBD, from human serum or plasma within as few as 8 min. The test showed a very high diagnostic sensitivity and specificity of 100% with 65 tested patient samples and thus performed better than two commercial tests for the same sample set. Additional advantages of the CoVRapid CL-MIA over the other test systems are the rapid analysis without extensive manual pipetting steps due to an automated flow-based principle of the assay. Due to this principle, the assay is more sensitive than common lateral flow “rapid tests” while still being very fast and easy to conduct without extensive manual steps in contrast to ELISA tests.

Due to the microarray principle, the simultaneous detection of antibodies to different antigens is possible with the CoVRapid CL-MIA, giving a more detailed insight into the individual immune response and diminishing the risk of false negative results. With our specialized microarray chip surface chemistry, we also achieved a negligibly small matrix influence that can be further reduced by on-chip matrix controls, enabling even the analysis of hemolytic blood samples.

With respect to the microarray chip production, also the covalent immobilization strategy for native proteins has to be emphasized in comparison to common assays that are based on the adsorption of denatured proteins. With native proteins, an environment comparable to the human cell is created, giving a realistic impression of the human immune response. Additionally, future adaption of the test for example by immobilization of antigens containing mutations is easily possible using the same antigen production and immobilization strategies as described herein.

This test is not only valuable in clinical surroundings to check whether a patient already overcame a SARS-CoV-2 infection and, especially, whether he still has antibodies that probably render him immune to fresh infection. It additionally can be very helpful in the upcoming time in connection with the SARS-CoV-2 vaccination that is already carried out in many countries and will be in the following months in many more. The test can be used to assess whether a vaccination has been successful and, hence, can aid in the control of vaccination status dependent admission criteria on-site.

Future research activities are planned to enlarge the scope of applications of the test. One aim is to transfer the microarray from glass to polycarbonate chips, making the fabrication even more economic. Additionally, the dual detection of IgM and IgG antibodies to SARS-CoV-2 and also to other respiratory viruses such as influenza will be expedited, as the flow-based concept is predestined for a two-step detection of different parameters. The detection of IgM antibodies furthermore would allow for a rapid diagnostic tool, e.g., in emergency rooms where patients with respiratory symptoms could be diagnosed rapidly after admission and subsequently be treated accordingly right from the beginning of their hospitalization. Another possible field of application would be a general vaccination monitoring for diseases such as measles, hepatitis A and B, or SARS-CoV-2 to allow for a rapid titer check by quantitative CL-MIA directly followed by vaccination if necessary.

Overall, we bring forward a valuable diagnostic tool that can easily be customized to different applications and already proved very successful in the context of SARS-CoV-2 serology testing.

## Electronic supplementary material

Supplementary Figure 1 Reaction schemes of applied immobilization strategies (PNG 228 kb)

High Resolution Image (EPS 124 kb)

(MP4 128 mb)

## Data Availability

Data will be made available upon reasonable request.
