# Supplementary figures and images for: Automated, flow-based chemiluminescence microarray immunoassay for the rapid multiplex detection of IgG antibodies to SARS-CoV-2 in human serum and plasma (CoVRapid CL-MIA)
Source: Anal Bioanal Chem. 2021 May 13;413(22):5619–32. doi: 10.1007/s00216-021-03315-6 (PMC8116441; doi:10.1007/s00216-021-03315-6)

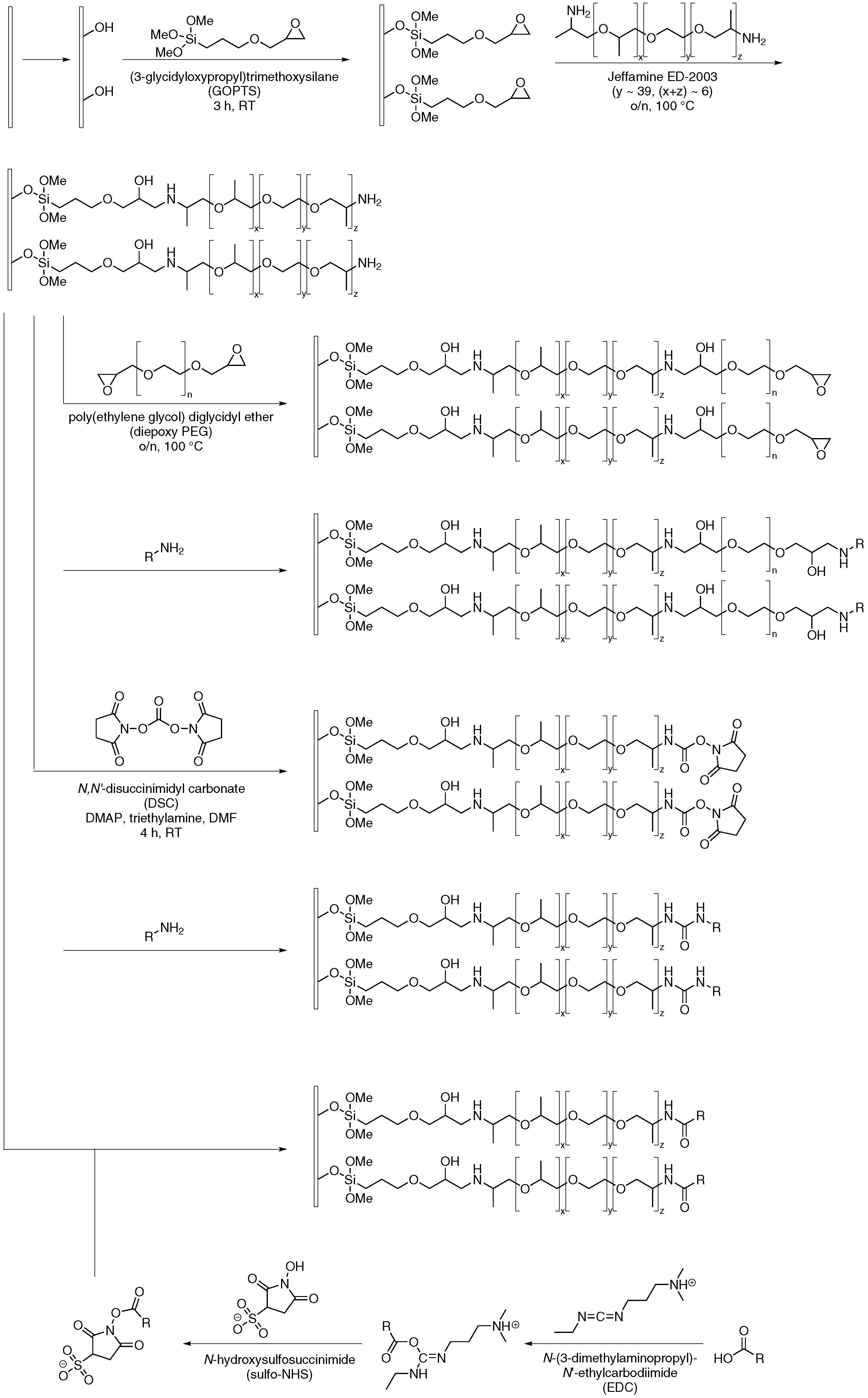

Supplement: Supplementary file 1 — Reaction schemes of applied immobilization strategies (PNG 228 kb) [file 216_2021_3315_Fig8_ESM.png]
